# The Zinc-Fingers of KREPA3 Are Essential for the Complete Editing of Mitochondrial mRNAs in *Trypanosoma brucei*

**DOI:** 10.1371/journal.pone.0008913

**Published:** 2010-01-27

**Authors:** Xuemin Guo, Nancy Lewis Ernst, Jason Carnes, Kenneth D. Stuart

**Affiliations:** Seattle Biomedical Research Institute, Seattle, Washington, United States of America; Emory University, United States of America

## Abstract

Most mitochondrial mRNAs in trypanosomes undergo uridine insertion/deletion editing that is catalyzed by ∼20S editosomes. The editosome component KREPA3 is essential for editosome structural integrity and its two zinc finger (ZF) motifs are essential for editing in vivo but not in vitro. KREPA3 function was further explored by examining the consequence of mutation of its N- and C- terminal ZFs (ZF1 and ZF2, respectively). Exclusively expressed myc-tagged KREPA3 with ZF2 mutation resulted in lower KREPA3 abundance and a relative increase in KREPA2 and KREL1 proteins. Detailed analysis of edited RNA products revealed the accumulation of partially edited mRNAs with less insertion editing compared to the partially edited mRNAs found in the cells with wild type KREPA3 expression. Mutation of ZF1 in TAP-tagged KREPA3 also resulted in accumulation of partially edited mRNAs that were shorter and only edited in the 3′-terminal editing region. Mutation of both ZFs essentially eliminated partially edited mRNA. The mutations did not affect gRNA abundance. These data indicate that both ZFs are essential for the progression of editing and perhaps its accuracy, which suggests that KREPA3 plays roles in the editing process via its ZFs interaction with editosome proteins and/or RNA substrates.

## Introduction

The mitochondrial DNA of trypanosomes, called kinetoplast DNA, is composed of tens of maxicircles and thousands of minicircles [1]. Each maxicircle DNA encodes two rRNAs and 18 proteins many of which are components of the oxidative phosphorylation system. Twelve maxicircle transcripts undergo the post-transcriptional uridine (U) insertion/deletion RNA editing to create the functional open reading frames (ORFs) [2], [3]. Some transcripts are extensively edited throughout their length, such as ATPase subunit 6 (A6) [4], cytochrome oxidase subunit III (COIII) [5], and ribosomal protein S12 (RPS12) [6]; while editing is restricted to smaller domains in other transcripts, such as apocytochrome b (CYb) [7], [8], cytochrome oxidase subunit II (COII) [9], and maxicircle unidentified reading frame 2 (MURF2) [10]. In addition, the editing of some mRNAs is developmentally regulated in the different life stages of trypanosomes. For example, little edited CYb and COII mRNAs are present in the mammalian bloodstream slender form (BF) stages of *T. brucei*, but substantial amounts of these edited RNAs are present in insect procyclic forms (PFs) [7], [9]. In addition, the 3′ domain of NADH dehydrogenase 7 (ND7) transcripts are only partially edited in BFs but fully edited in PFs [11].

The editing sites and the number of inserted/deleted U's are specified by guide RNAs (gRNAs), almost all of which are encoded in minicircle DNA with a few encoded in maxicircle DNA [12]–[14]. RNA editing proceeds in the 3′ to 5′ direction and initiates by the formation of an anchor duplex between pre-mRNA and its cognate gRNA, with the editing sites (ESs) upstream (5′ relative to mRNA) of the anchor duplex [15], [16]. One cycle of RNA editing entails a series of enzymatic reactions that include endonucleolytic cleavage, U addition or removal, and RNA ligation as specified by the gRNA. Multi-protein complexes, the ∼20S editosomes, contain the enzyme activities for these steps and can catalyze a full round of RNA editing cycle in vitro [17], [18]. In our current understanding, deletion ESs are cleaved by editosomes that contain endonuclease KREN1, while insertion ESs are cleaved by editosomes that contain KREN2 or KREN3 endonuclease [19]–[22]. Subsequent to cleavage, one or more Us are either removed or added from the 3′ end of the 5′ mRNA fragment by a U specific exonuclease (KREX1 or KREX2) or the 3′ terminal uridylyl transferase (KRET2), respectively [23]–[26]. The processed 5′ fragment is then religated with the 3′ fragment by RNA ligase KREL1 or KREL2 [27], [28]. All of these steps are specified by the gRNAs which interact with their cognate mRNAs. Most pre-mRNAs require multiple cycles of RNA editing and multiple gRNAs to become functional. However, little is known about the succession of editing from one ES and from one gRNA to the next. The KREN1, KREN2, or KREN3 endonucleases along with one or two other proteins are unique to three different ∼20S editosomes that contain a common set of twelve proteins [20], [22]. The interactions among these different editosomes and the mechanism that they employ to recognize their respective processing sites remains to be determined.

In addition to ∼20S editosomes, other complexes have been shown to be important for editing including those that contain RET1, MRP1/2, RBP16, or TbRGG1 and TbRGG2 [29]–[37]. Their roles may involve the binding, pre-processing, and transporting of the gRNAs and mRNAs. RNAi knockdown of these proteins has various effects on the levels of different edited and pre-edited mRNAs. TbRGG2 knockdowns lead to a dramatic decrease of extensively edited RNAs and moderate stabilization of never-edited and minimally edited RNAs [32]. RBP16 and MRP1/2 showed the similar effect on the editing and stability of some RNAs but function distinctly or reduntantly depending on the RNAs. Each knockdown resulted in reduced levels of never edited ND4 and COI RNAs and a dramatic decrease in edited CYb RNA but no effect on the editing of most of the extensive edited mRNAs [17], [38], [39]. Simultaneous depletion of RBP16 and MRP1/2 resulted in additive inhibition effect on edited CYb RNA level but not on ND4 and COI RNA levels, and dramatically reduced the extensive edited A6 and COIII RNA levels [40]. The functional and physical associations between these complexes and ∼20S editosomes are unclear. Knockdown of components of any one of these complexes has little effect on the integrity or abundance of the other complexes showing that their presence is not interdependent [41]. Gradient sedimentation studies show RNase sensitive associations between some of these complexes but it is not yet known if these occur in vivo or result from experimental manipulation [30], [42]–[44]. Thus, these complexes have roles related to editing and they may associate, if only transiently, but their specific roles and how they differentially affect the abundance of edited, pre-edited, and never edited mRNAs are yet to be elucidated.

The ∼20S editosomes contain many proteins of which most are inter-related in sets or pairs based on their amino acid sequences and predicted functional domains [45]. A general structural and functional organization is emerging in which insertion and deletion editing appears physically and functionally separated in two distinct heterotrimeric subcomplexes [18], [46]. In addition, there are three types of these ∼20S editosomes, each with a different endonuclease along with one or two partner proteins and distinct ES cleavage specificity [19]–[22]. All three editosomes have an identical “core” of 12 proteins that is composed of the two distinct heterotrimeric subcomplexes, four related OB-fold proteins (KREPA3, 4, 5, and 6), and two related proteins with degenerate RNase III motifs (KREPB4 and 5). The insertion heterotrimeric subcomplex contains the KRET2 3′TUTase linked by KREPA1 to KREL2 RNA ligase. The deletion heterotrimeric subcomplex contains the KREX2 U specific 3′ exoUase linked by KREPA2 to KREL1 RNA ligase. The binary interactions of KREPA1 and KREPA2 with their specific enzymes enhance the catalytic activity of the latter [46]. The KREPA3, 4, 5 and 6 proteins each have a C-terminal OB-fold domain, as do KREPA1 and KREPA2. KREPA1, 2, and 3 also have two C2H2 ZF motifs, which share a similar pattern of (F/T)XCX2CX3FX5ΨX2HX4H and may function in protein-protein interaction or RNA-binding as may the OB fold motif domains [45], [47]. Knockdowns of KREP A3, 4, 6 (A5 has not been tested), and KREPB4 and 5 results in essentially complete loss of the ∼20S editosomes, indicating that these proteins are essential for editosome integrity and stability [48]–[54]. While KREPA proteins may have no catalytic function, the degenerate RNAse III motifs of KREPB4 and 5 may function in association with the KREN1, 2, or 3 endonucleases.

The specific role of KREPA3 is uncertain. RNAi knockdowns of KREPA3 in PFs lead to partial disruption of the ∼20S editosomes and loss of endonuclease activity, but retention of the U addition, U removal, and RNA ligase activities that are characteristic of the insertion and deletion subcomplexes [49], [51]. The more extensive repression of KREPA3 expression in T. brucei BFs by regulatable knockout (RKO) leads to essentially complete loss of ∼20S editosomes as well as in vitro and in vivo RNA editing activity [49]. It is uncertain if the difference between the results in PFs and BFs reflects differences between the life cycle stages or the degree of knockdown. The significance of the endo- and exo-nuclease activity found with recombinant KREPA3 is uncertain but the enhancement or restoration of endo-and exo-nuclease activities upon addition of this protein to KREPA3 depleted editosomes implies a restoration of functional architecture [49], [51], [55]. Exclusive expression of TAP-tagged KREPA3 with mutated ZF motifs or deleted OB-fold domain revealed that the ZF motifs are essential for growth and RNA editing in vivo but not for protein interaction. However, the OB-fold domain is necessary for protein interaction since its absence results in loss of editosomes and subcomplexes [49]. Hence KREPA3 may have dual roles in editosome architecture and RNA binding and function in coordinating the steps of editing.

In this paper, we further explore the consequences of disrupting the ZF motifs of KREPA3 on in vivo RNA editing. We find that, in contrast to the TAP tag, the C-myc tag has no effect on KREPA3 function in RNA editing in vivo or incorporation into ∼20S editosomes. C-myc tagging allowed further analysis of the effects of ZF mutations of KREPA3 on RNA editing and editosome integrity. Loss of KREPA3 protein or editosomes had no effect on total gRNA abundance. However, exclusive expression of KREPA3 ZF mutants shows that the both ZF motifs are essential for editing progression in vivo, and based on the analysis of RT-PCR products of edited mRNAs, each ZF appears to play a distinct functional role in editing.

## Results

**Figure 1:**
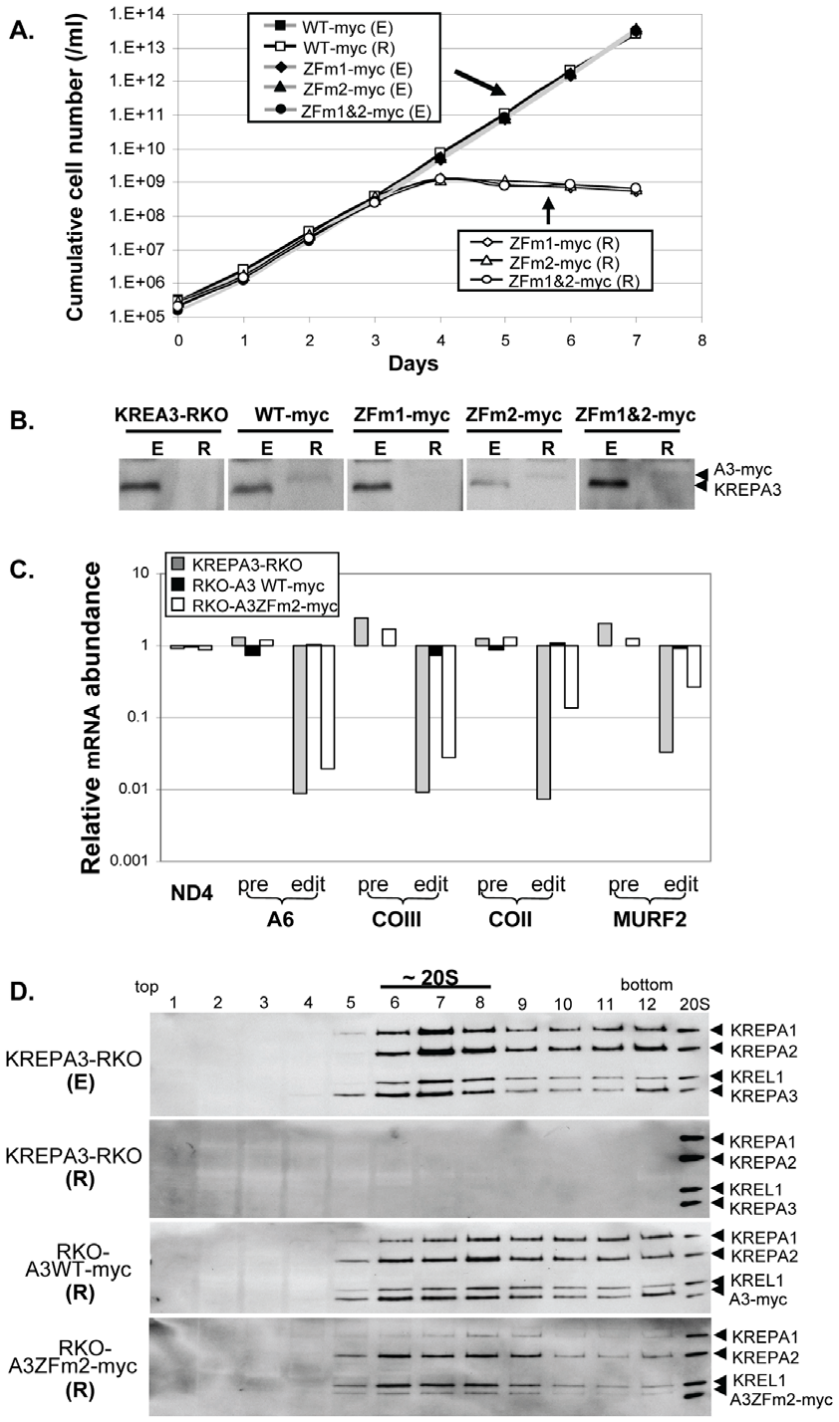
Effects of ZF mutation of KREPA3 with a myc-tag on growth, editing, and editosome integrity. C-terminal myc-tagged WT KREPA3 protein or KREPA3 with mutated ZF1, ZF2, or ZF1&2 were constitutively expressed from the β-tubulin locus and were exclusively expressed upon repression of KREPA3 Reg allele expression by tet withdrawal. (**A**) Growth of RKO-KREPA3 WT-myc, ZFm1-myc, ZFm2-myc, and ZFm1&2-myc cell lines in which KREPA3 Reg was expressed (E) (grey line and solid symbol) or repressed (R) (black line and open symbol). Only WT-myc cells survive after KREPA3 Reg is repressed. (**B**) Western analysis using KREPA3-specific MAb to probe samples from the cells in panel A with KREPA3 Reg expressed (E) or repressed (R) for three days; parental cell line KREPA3-RKO is used as a control. (**C**) Real time PCR analysis of in vivo RNA editing in RKO-A3 WT-myc or ZFm2-myc cells in which KREPA3 Reg was repressed for three days. The relative amounts of pre-edited and edited mRNAs A6, COIII, COII, and MURF2 and never-edited ND4 mRNAs was normalized to 18S rRNA and compared to the corresponding cells in which KREPA3 Reg was expressed. The same analysis was done with KREPA3-RKO cells as a control. Note the log scale and that 1 represents no difference, >1 an increase, and <1 a decrease in relative RNA amount. (**D**) Western analysis of the glycerol gradient fractions of crude mitochondrial lysates from RKO-KREPA3 cells in which KREPA3 Reg was expressed (E) or repressed for three days (R) or from RKO-A3 WT-myc or ZFm2-myc cell in which myc tagged WT or ZF2 mutated KREPA3 was exclusively expressed, respectively, by three days repression (R). The analyses used a mixture of MAbs specific for the KREPA1, KREPA2, KREL1, and KREPA3 ∼20S editosome proteins. Purified ∼20S editosomes used as a control show the size of the untagged KREPA3 protein.

The two C2H2 ZF of KREPA3 are located at the 5′ part of the ORF and widely separated by 108 amino acids, The ZF mutants were constructed by replacing the two cysteines with alanines in each or both ZFs as previously described [49]. Cell lines that exclusively express normal or mutated KREPA3 were generated in order to explore ZF function [49]. The genetic background for conditional exclusive expression of WT or mutated KREPA3 was provided by the KREPA3-RKO cell line in which both endogenous KREPA3 alleles have been eliminated and a tet-regulated WT allele (KREPA3 Reg) has been inserted into the rDNA intergenic locus. After insertion of a WT or mutated KREPA3 gene into the β-tubulin locus of KREPA3-RKO cells (where it is constitutively expressed), withdrawal of tet represses expression of KREPA3 Reg allele and thus results in cells that only express the KREPA3 allele in the β-tubulin locus. Each KREPA3 allele was C-myc tagged to monitor expression as well as the incorporation into and the effect on editosomes, since previous studies showed that KREPA3 with a larger C-terminal TAP tag inhibited growth, disrupted or destabilized editosomes, and diminished in vivo RNA editing [49]. The resulting cells were designated RKO-A3WT-myc, RKO-A3ZFm1-myc, RKO-A3ZFm2myc, and RKO-A3ZFm1&2-myc depending on whether the allele in the tubulin locus was mutated in the N-terminal (ZFm1), C-terminal (ZFm2), or both ZFs (ZFm1&2). All of these cells grew normally for 7 days in the presence of tet, i.e. when KREPA3 Reg was expressed (E) (Fig. 1A). Cells expressing KREPA3WT-myc also grew normally upon repression (R) of KREPA3 Reg by withdrawal of tet, showing that exclusive expression of myc tagged KREPA3 does not affect growth in contrast to TAP tagged KREPA3. However, cells expressing KREPA3-myc with one or both ZFs mutated ceased growing by day four following tet withdrawal. Western analysis of the cell samples with KREPA3 monoclonal antibody (MAb) showed that the myc-tagged WT and A3ZFm2 were only detected after the expression of untagged KREPA3 Reg was repressed (R), and their apparent protein levels were lower than that of KREPA3 Reg; neither A3ZFm1 nor A3ZFm1&2 proteins were detected after repression of KREPA3 Reg expression (R) (Fig. 1B). In contrast, the levels of TAP-tagged KREPA3 WT and ZF2 mutant proteins were similar to that of untagged KREPA3 and their expression was not affected by the presence of the untagged protein [49]. Our data show that substantially reduced amounts of KREPA3 can support normal cell growth and RNA editing (Fig. S1) although we cannot exclude the possibility that the death of RKO-A3ZFm1-myc and RKO-A3ZFm1&2-myc cells is due to an insufficient amounts of these mutated proteins upon KREPA3 Reg repression. Since the small amount of myc-tagged A3ZFm1 and ZFm1&2 in cells exclusively expressing these proteins prevented clear analysis of KREPA3 function, we focused the studies on the RKO-A3ZFm2-myc cell line to determine the effect of this ZF mutation on RNA editing and the editosome integrity. While the C-terminal myc-tag on KREPA3 affects protein abundance, it does not affect growth, and because the lower amount of KREPA3 is enough to support normal growth, the function of ZF2 is essential for growth.

Analysis of edited, pre-edited, and never edited mRNA levels by real-time PCR revealed that mutation of ZF2 decreases editing in vivo. Exclusive expression of A3WT-myc for three days had no effect on the abundance of edited, pre-edited, or never edited mRNAs compared to the cells in which the KREPA3 Reg allele was expressed from the rDNA intergenic region. Hence the myc tag had no detectable effect on editing in these real-time PCR studies of mt mRNAs normalized to 18S rRNA (Fig. 1C). This is in contrast to the essentially complete loss of edited, but not pre-edited, mRNAs upon repression of KREPA3 Reg expression in the RKO cells. Exclusive expression of A3ZFm2-myc for 3 days resulted in a dramatic reduction of A6 and COIII edited mRNAs and a substantial reduction of edited COII and MURF2 mRNAs, but little or no change in their pre-edited RNAs. Hence, the function of ZF2 is critical for editing in vivo since the effect of its disruption is similar to that resulting from the loss of editosomes.

**Figure 2:**
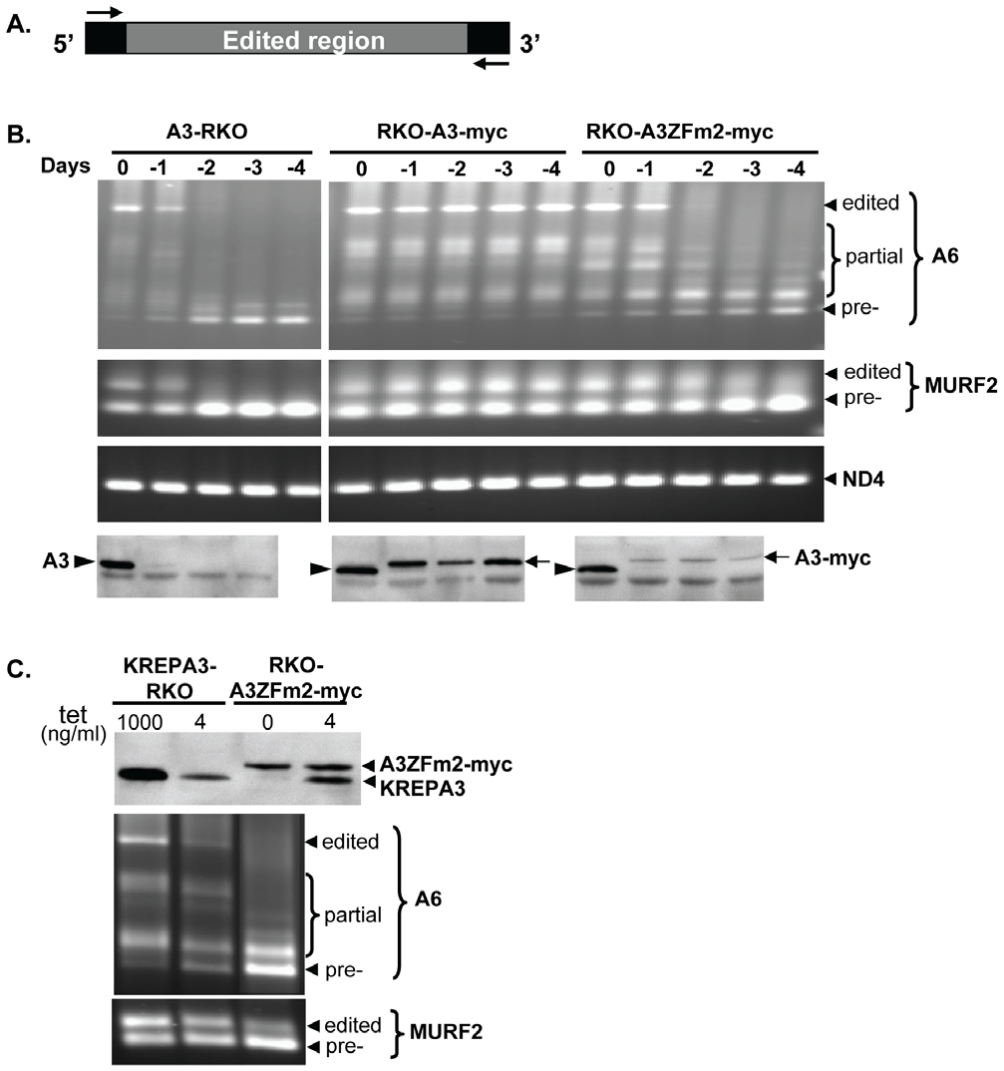
Partially edited A6 and MURF2 mRNAs accumulate in cells exclusively expressing KREPA3ZFm2. (**A**) Schematic showing the unedited flanking sequences used as the upstream/downstream primers for RT-PCR. (**B**) Agarose gel analysis of the RT-PCR products of A6 and MURF2 mRNAs following 1 to 4 days repression of KREPA3 Reg expression in KREPA3-RKO, RKO-A3 WT-myc and ZFm2-myc cells. The locations of the edited, partially edited, and pre-edited cDNA bands are indicated. Never-edited ND4 mRNA was used as a control. Expression of untagged and myc-tagged KREPA3 protein was monitored by Western analyses using a MAb specific for KREPA3 (lowest panel). (**C**) Comparison of the protein level of exclusively expressed KREPA3ZFm2-myc in RKO-A3 ZFm2-myc cells to the untagged KREPA3 in RKO-KREPA3 cells induced with 4 ng/ml tet by Western analysis using KREPA3 specific MAb (top panel) and agarose gel analysis of the corresponding RT-PCR products of A6 and MURF2 mRNAs (lower panel).

Western analyses of glycerol gradient fractions, using an antibody mix that detects four editosome proteins, showed that myc tagged WT and ZF2 mutated KREPA3 were incorporated into ∼20S editosomes (Fig. 1D). Analysis of crude mitochondrial lysates from the parental KREPA3-RKO cell line that is expressing KREPA3 Reg (E) shows the typical sedimentation profile with a peak at ∼20S and some material at higher S value, while repression of KREPA3 Reg expression for 3 days (R) results in essentially complete loss of editosomes in this parental cell line. Editosomes are present in cells that exclusively express either the KREPA3 WT-myc or ZFm2-myc alleles from the –tubulin locus, and the sedimentation profiles of the editosomes are essentially the same as that in the KREPA3-RKO (E). The amount of KREPA3ZFm2-containing editosomes appears to be lower than that of WT-myc-containing editosomes as a result of the lower level of ZFm2-myc (Fig. 1D, also seen Fig. 2B and C). The ratio of editosome proteins in the WT-myc-containing editosomes detected by western analysis was similar to that in those from KREPA3-RKO (E) cells. Thus myc tag appears to have no effect on the incorporation of KREPA3 into editosomes. However, the levels of KREPA2 and KREL1 appear increased relative to KREPA1 and A3ZFm2-myc in editosomes rescued by A3ZFm2-myc, which is consistent with a previous observation using co-immunoprecipitation of TAP-tagged KREPA3 ZF mutants [49]. This suggests that the KREPA3 ZF2 motif affects editosome stability and/or assembly. Overall, these results show that the myc tag has no effect on cell growth, RNA editing, or KREPA3 incorporation into ∼20S editosomes. The results further indicate that KREPA3 ZF motif is essential for cell viability and in vivo editing. The decrease in vivo editing activity is disproportionate to the decrease in editosome abundance in RKO-A3ZFm2-myc cells, implying that the loss of viability and editing is due to editosome dysfunction.

### Mutation of the ZF2 of KREPA3 Affects Editing Progression In Vivo

To examine the effect of the ZF2 mutation on editing in vivo, we analyzed ATPase subunit 6 (A6) and MURF2 editing after exclusive expression of KREPA3ZFm2-myc. Mt RNAs were amplified by RT-PCR of total cellular RNA from KREPA3-RKO and derived cell lines that exclusively express myc-tagged KREPA3WT or ZFm2. These studies used primers that non-selectively amplify edited and unedited A6 and MURF2 mRNAs (Fig. 2A) as well as ND4 mRNA that does not get edited. The products were generated from KREPA3-RKO, RKO-A3WT-myc and RKO-A3ZFm2-myc cells in which KREPA3 Reg was expressed or repressed for various lengths of time and examined by gel electrophoresis (Fig. 2B). Edited A6 and MURF2 mRNA levels were decreased after one day of KREPA3 Reg repression in KREPA3-RKO cells and were undetectable after two days, while their pre-edited counterparts accumulated. Correspondingly, western analysis showed that the KREPA3 protein level was dramatically reduced in the absence of tet for one day and was not detectable after two days later. Repression of KREPA3 Reg allele expression in RKO-KREPA3 WT-myc cells had no effect on the levels of edited or pre-edited A6 and MURF2 mRNAs despite the replacement of KREPA3 protein with KREPA3-myc. Hence exclusive expression of myc-tagged KREPA3 WT can support the normal editing in vivo. In contrast, repression of KREPA3 Reg allele expression in RKO-A3ZFm2-myc cells resulted in the severe depletion of fully edited A6 mRNA and the accumulation of pre-edited and some partially edited (white arrow) A6 mRNAs. Exclusive expression of A3ZFm2-myc also resulted in the loss of edited MURF2 mRNA and the accumulation of pre-edited and partially edited MURF2 mRNA, although this is less obvious since the region of MURF2 that is edited is much smaller than that of A6. Never-edited ND4 mRNA was unaffected upon KREPA3 Reg repression in all cell lines. Overall, these results suggest that editosomes containing KREPA3 with mutated ZF2 can edit in vivo but they are not fully functional.

Because the amount of A3ZFm2-myc protein was lower than A3WT-myc after repression of the KREPA3 Reg allele, we investigated whether the reduced amount of KREPA3 alone could account for the observed phenotype following exclusive expression of A3ZFm2-myc (Figs. 2B and C). To assess whether this lower amount of KREPA3 was responsible for the loss of fully edited mRNAs and the accumulation of some partially edited mRNAs, the expression of untagged KREPA3 Reg allele was adjusted in RKO-KREPA3 cells by reducing tet concentration. Growth was progressively inhibited at tet concentrations below 3 ng/ml, a level at which the cells grew normally and the amounts of KREPA3 protein and fully edited A6 and MURF2 mRNAs were readily detectable (Fig. S1). Western analysis of cells grown in 4 ng/ml tet showed that untagged KREPA3 protein was reduced in the KREPA3-RKO cells compared to the normal induction with 1000 ng/ml (Fig. 2C top panel). This reduced amount of KREPA3 was similar to the amount of the constitutively expressed KREPA3ZFm2-myc protein in RKO-A3ZFm2-myc cells grown in either 0 or 4 ng/ml of tet (Fig. 2C top panel). RT-PCR analysis (Fig. 2C bottom panel) revealed that KREPA3-RKO cells grown in 4 ng/ml tet contained normally edited A6 and MURF2 mRNAs that, although at reduced levels, were sufficient to support normal cell growth. The pre-edited amounts of these mRNAs were also increased slightly. In contrast, in cells exclusively expressing A3ZFm2-myc the fully edited A6 mRNA was essentially absent while partially edited and pre-edited A6 mRNA accumulated; edited MURF2 mRNA was similarly diminished and partially edited and pre-edited MURF2 mRNA accumulated, despite the amount of KREPA3ZFm2-myc being similar to that of untagged KREPA3. Thus, the mutation of ZF2 in KREPA3, rather than its lower amount, is responsible for the loss of edited mRNAs and the accumulation of some partially edited and pre-edited mRNAs.

**Figure 3 pone-0008913-g003:**
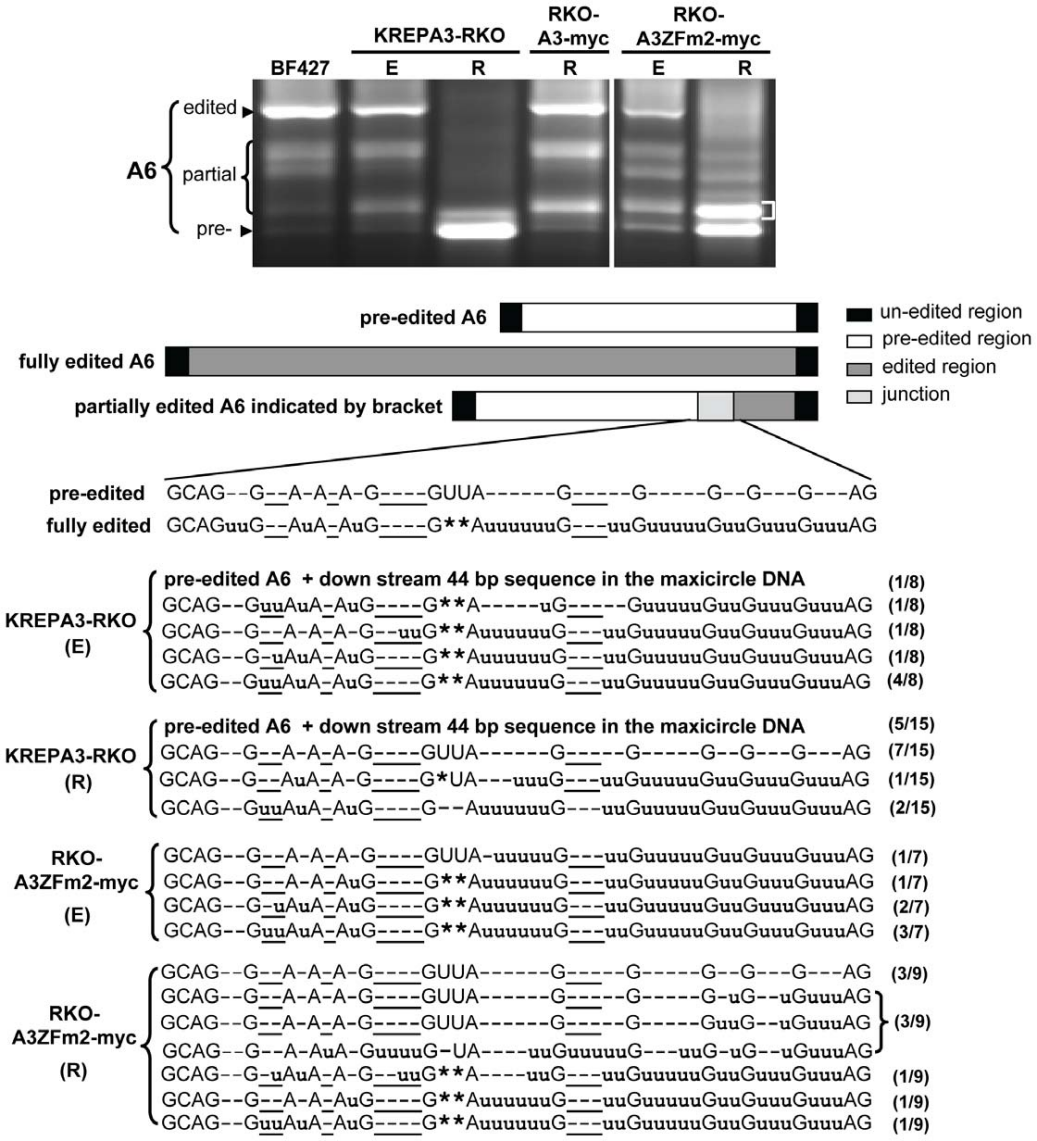
Sequences of gel isolated RT-PCR products of the partially edited A6 mRNAs. A6 mRNA RT-PCR products from KREPA3-RKO and RKO-A3ZFm2-myc cell with KREPA3 Reg expressed (E) or repressed for three days (R), respectively, were isolated from agarose gels. BF 427 RNA was used as a control. The region of the gel from which the DNA was cut, cloned and sequenced is indicated with the bracket. The relative location of the partially edited sequence in the junction between the unedited and edited sequences is shown schematically. Inserted u's are shown in lower case, each deleted U is shown as an asterisk (*), and those not matching fully edited RNA are underlined. The number of the clones found with each shown sequence is indicated in parentheses on the right. In some cases (indicated by text instead of sequence), pre-edited A6 sequences were obtained due to mis-priming at a site downstream of the A6 coding sequence, thereby creating PCR products large enough to be found in the excised band.

Exclusive expression of A3ZFm2-myc resulted in the significant accumulation of a partially edited A6 band in RT-PCR analyses (labeled with a white bracket in top panel of Fig 3). To characterize this major band corresponding to the partially edited A6 mRNA, it was cut from the gel, cloned, and sequenced from the lanes of both KREPA3-RKO and RKO-A3ZFm2-myc cells with KREPA3 Reg expressed (E) or repressed (R) for 3 days. Most of the sequenced cDNAs were derived from partially edited mRNAs with incomplete editing in the junction between the fully edited and pre-edited regions (Fig. 3 bottom panel). Except for the occasional pre-edited precursor clone, most clones from RKO-KREPA3 cells (E) were partially edited, including some ‘non-canonical’ editing that was also found in WT BF 427 cells (data not shown). Such ‘non-canonical’ editing does not match the pre-edited or fully edited mRNA, and has previously been observed in normal cells [56]–[58]. The clones from KREPA3-RKO cells after KREPA3 Reg has been repressed for 3 days had similar sequence characteristics, except that most of the clones had pre-edited sequences, consistent with the loss of the editing activity. When the KREPA3 Reg allele was expressed (E), the sequences from the RKO-A3ZFm2-myc cells were similar to those from KREPA3-RKO cells. In contrast, cells that exclusively express A3ZFm2-myc [RKO-A3ZFm2-myc (R)] had a greater number of clones with pre-edited sequences, a greater proportion of the clones containing less insertion editing in the junction region, and fewer edited sequences than in control cells with KREPA3 Reg expressed. Overall, these sequence analyses indicate that editosomes with a mutation in the C-terminal ZF of KREPA3 can edit in vivo, but the progression of editing is severely inhibited, resulting in the accumulation of partially edited A6 RNAs.

**Figure 4 pone-0008913-g004:**
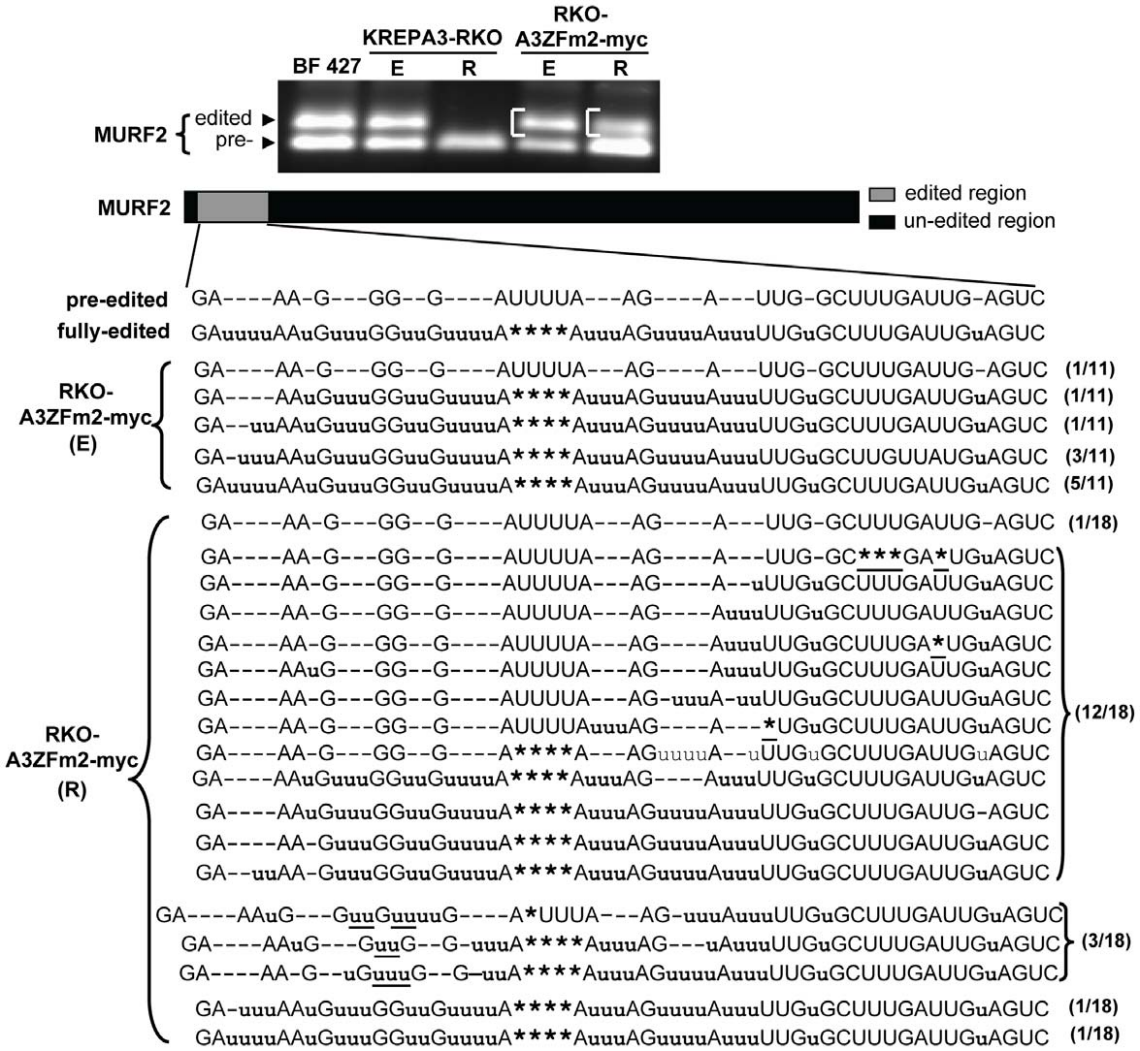
Sequences of gel isolated RT-PCR products of the edited MURF2 mRNAs. The edited MURF2 RT-PCR products (indicated by brackets) from RKO-KREPA3ZFm2-myc cells with KREPA3 Reg expressed (E) and repressed (R) were cut from agarose gel, cloned and sequenced. The inserted, deleted, and non-matching positions and numbers of sequenced clones are indicated as in Figure 3.

To determine if the in vivo editing deficiency observed in partially edited A6 sequences was also present in other edited RNAs, MURF2 in vivo editing was assessed in RKO-A3ZFm2-myc cells. MURF2 PCR products larger than the pre-edited product were cloned and sequenced from RKO-A3ZFm2-myc cells with KREPA3 Reg expressed (E) or repressed (R) (Fig. 4). Editing of the most 3′ single U insertion can be directed by gMURF2-I, while the more 5′ block can be directed by gMURF2-II (Fig. S2) [59], [60]. Other unknown gRNAs for MURF2 may exist. Of eleven sequenced MURF2 clones from the cells in which KREPA3 Reg was expressed, ten were fully edited to a translatable RNA and one was pre-edited. In contrast, of 18 sequenced clones from RKO-A3ZFm2-myc (R) cells, only two clones were fully edited to translatable mRNAs. The remaining clones had varying degrees of incomplete editing. Nine did not have Us completely removed from the deletion editing site, and editing in these clones was generally restricted to the region 3′ to this deletion site. The remaining clones were edited 5′ to the deletion site. In addition, some ‘non-canonical’ editing was only found in sequences from cells in which KREPA3ZFm2 was exclusively expressed. These results further indicate that the ZF2 of KREPA3 is essential for editing progression, and mutation of ZF2 of KREPA3 inhibited the completion of editing in vivo as well as increasing the proportion of ‘non-canonical’ editing. It cannot be concluded whether these results are due directly to mutation of ZF2 and hence KREPA3 function, or altered editosome structure, or other factors.

### The ZF1 of KREPA3 Is Also Essential for the Editing Progression In Vivo

**Figure 5:**
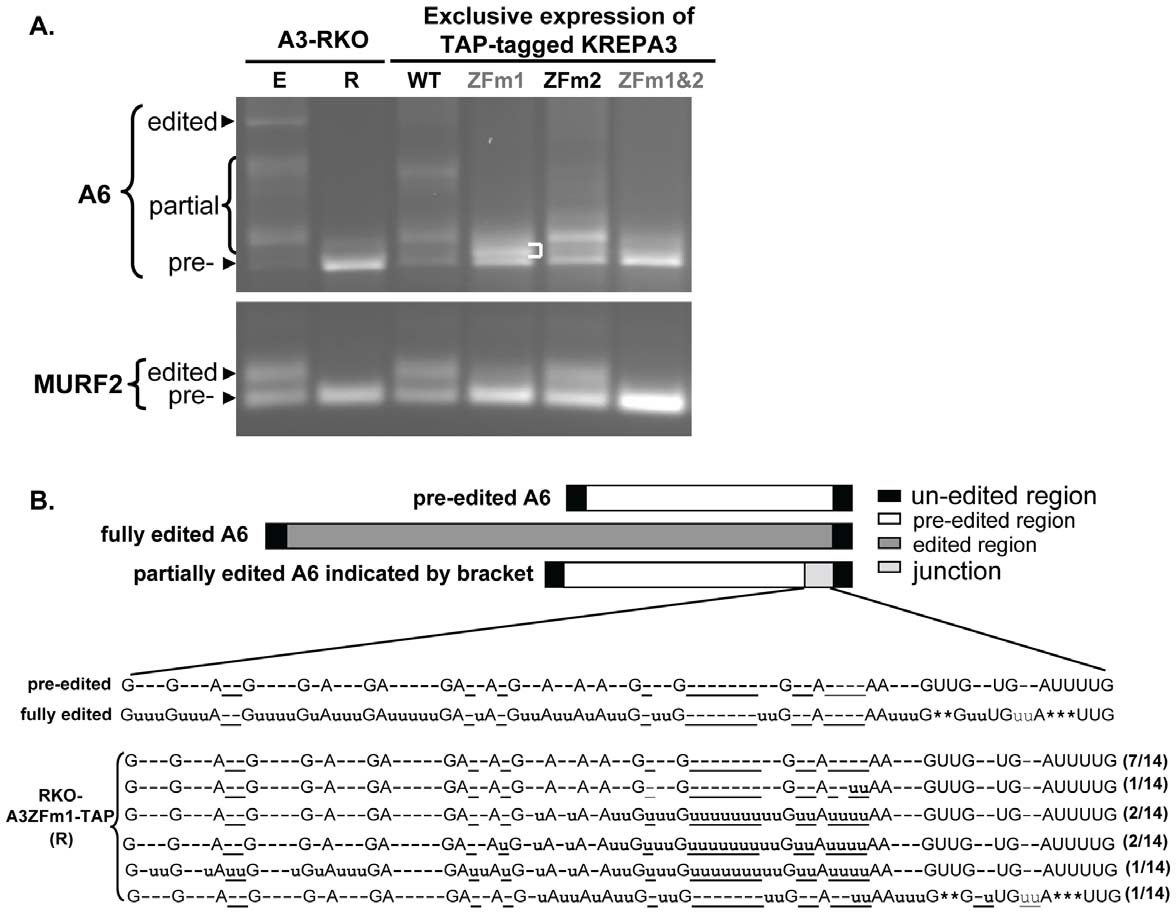
Effect of ZF1 mutation in KREPA3 on RNA editing progression. (**A**) Agarose gel analysis of A6 and MURF2 mRNAs RT-PCR products from cells that exclusively expressed WT TAP-tagged KREPA3 or mutants with one or both ZFs mutated (see [49]). RT-PCR products from KREPA3-RKO cells with KREPA3 expressed (E) or repressed (R) were used as controls. (**B**). Sequence analysis of gel isolated partially edited A6 mRNAs that accumulated (indicated by bracket) upon mutation of KREPA3 ZF1. The location of the partially edited junction is shown schematically. Sequence designations as in Figures 3 and 4.

Both ZF1 and ZF2 of KREPA3 were shown to be essential for the RNA editing in vivo, but were not required for editosome catalytic activity in vitro [49]. We performed RT-PCR to assess the effect of the mutation of ZF1 on editing progression. The cellular levels of KREPA3ZFm1-myc and ZFm1&2-myc is much lower than that of ZFm2-myc, but TAP-tagged KREPA3ZFm1 and ZFm1&2 express at a higher level than do the myc-tagged versions as described above. Although the TAP tag has an effect on RNA editing in vivo, KREPA3ZFm2-TAP inhibited A6 editing similar to that of KREPA3ZFm2-myc as revealed by RT-PCR (Fig. S3) and by sequence analysis (data not shown). Thus we chose the TAP tagged RKO-KREPA3 WT and ZF mutants cell to study the effect of the ZF1 mutation on the editing process in vivo. Total RNAs were harvested from KREPA3-RKO derived cells that exclusively express TAP-tagged KREPA3 WT and mutants with one or both ZFs mutated and RT-PCR was performed to amplify A6 and MURF2 mRNAs (Fig. 5A). As before, KREPA3ZFm2 reduced the level of fully edited RNA and resulted in the accumulation of partially edited A6 and MURF2 RNAs. KREPA3ZFm1 also resulted in the accumulation of partially edited A6 mRNAs, but these are obviously smaller (i.e. have undergone less editing) than those accumulating in the RKO-KREPA3ZFm2-TAP cells. The amount of partially edited MURF2 mRNA was similarly decreased, and the products shorter in KREPA3ZFm1 EE cells compared to KREPA3ZFm2 EE cells. Very little partially edited A6 and essentially no edited MURF2 RNAs were found in RKO-A3ZFm1&2-TAP cells, similar to what was observed in KREPA3-RKO cells in which KREPA3 Reg was repressed. These results indicate that both the ZF1 and ZF2 of KREPA3 are necessary for editing progression.

To characterize the editing deficiency caused by ZF1 mutation, we analyzed the sequences of partially edited A6 mRNAs that accumulated in RKO-A3ZFm1-TAP EE cells (Fig. 5). These products were smaller than those obtained when ZF2 was mutated. The major band of PCR products that correspond to partially edited A6 mRNA (indicated with brackets) was excised, cloned and sequenced (Fig. 5B). Half of the 14 sequenced clones were pre-edited, which may reflect the similar sizes of the pre-edited products and the major band. The remaining sequenced products were partially edited at the 3′-terminus adjacent to the unedited region. The position and size of this region could be specified by a single gRNA. All seven of these clones have one or more uridines inserted at ‘non-canonical’ sites that are not present in fully edited mRNA, and all but one of these lack editing at the 3′ terminal five ‘canonical’ editing sites, which include both insertion and deletion sites. The characteristics of the accumulated partially edited products in KREPA3ZFm1 EE cell are distinct from the ones in KREPA3ZFm2 EE cell. These data indicate that both the zinc fingers are essential for complete editing and hence editing progression, but their functions are different.

### KREPA3 Loss or ZF Mutation Does Not Affect Total gRNA Abundance

**Figure 6:**
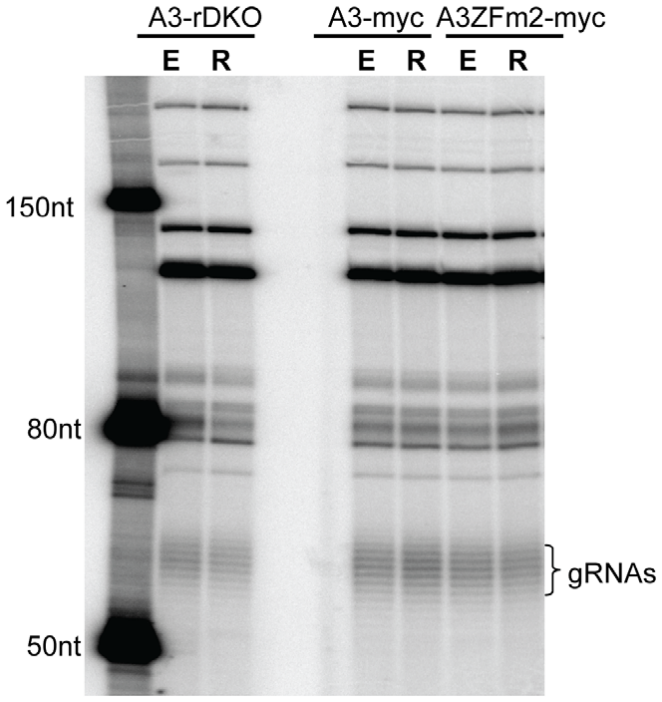
Loss of KREPA3 or mutation of KREPA3 ZF2 has no effect on gRNA levels. Total cellular RNA from KREPA3-RKO, RKO-KREPA3 WT-myc and ZFm2-myc cells with KREP3 Reg expressed (E) or repressed (R) were examined by a capping assay using guanylyltransferse and [α-^32^P]GTP. The similarly labeled ssRNA ladder was used to size the gRNAs which show their characteristics size heterogeneity due to their variable oligo-U tails.

Since real-time PCR analysis showed that the loss of KREPA3 or mutation of its zinc fingers blocks editing of multiple mRNAs, we explored whether this could be related to the production of the gRNAs. Total RNAs were harvested from KREPA3-RKO and derived cell lines RKO-A3WT-myc and ZFm2-myc with KREPA3 Reg expressed (E) or repressed (R), respectively. The total gRNA levels were assessed by 5′ labeling the gRNAs using α-[^32^P]GTP and guanylytransferase (Fig. 6). The levels and sizes of the gRNAs from all of these cells were essentially indistinguishable. This indicates that neither the loss of KREPA3 and the subsequent loss of ∼20S editosomes after KREPA3 Reg repression, nor the altered function due to ZF mutation had an effect on total gRNA levels. This also suggests that ∼20S editosomes are not required for the processing and maturation of gRNAs.

## Discussion

This study shows that ZFs of KREPA3 are important for editosome function in vivo. Exclusive expression of myc-tagged KREPA3 with one or both ZFs mutated inhibits both growth and editing in vivo (Fig. 1A and C), similar to previously described TAP-tagged KREPA3 ZF mutants [49]. Importantly, some partially edited mRNAs accumulate in the cells that exclusively express KREPA3 with a mutated ZF (Fig. 2B and C, Fig. 5A). A subset of partially edited sequences (Fig. 3–5) contain ‘non-canonical’ editing that does not match the sequence of fully edited mRNA, and resembles sequences sometimes observed in wild type cells [56]–[58], Noticeably, the frequency of observing these ‘non-canonical’ editing products significantly increases after mutation of KREPA3 zinc fingers. We cannot exclude the possibility that mutation of the ZFs alters the precision of editing. Thus, these data demonstrate that editosomes containing KREPA3 with mutant ZF can perform RNA editing in vivo but are dysfunctional, and KREPA3 is critical for the complete progression of editing, either through direct involvement in the editing process or indirect effect on editosome structure. KREPA3 with mutation of ZF1, which could only be examined in cells exclusively expressing the TAP-tagged KREPA3ZFm1 [49], resulted in accumulation of partially edited ATP synthase 6 mRNA in which the editing was restricted to the most 3′ region that could be specified by a single gRNA (Fig. 5). However, exclusive expression of TAP-tagged KREPA3ZFm2 resulted in accumulation of partially edited ATP synthase 6 mRNA that was more extensively edited and required the use of multiple gRNAs; similar results were obtained from cells exclusively expressing this mutant KREPA3 with myc-tag (Fig. 4 and Fig. S3). Therefore, in the context of the TAP-tag, the impact of mutation of ZF1 was more severe than that of ZF2. The defect in editing progression caused by ZF mutation of KREPA3 is not due to gRNA abundance, since mutation of ZF1 and/or ZF2 did not affect the levels of gRNAs (Fig. 6); however, gRNA utilization might be affected. The possible roles of KREPA3 include structurally organizing the editosome proteins and hence orienting editosome catalysts and RNA binding and/or possibly coordinating the multiple steps of editing, which must entail considerable molecular movement. Our data also indicate that KREPA3 is important for editsome stability. KREPA3 with a myc tag incorporates into editosomes but the abundance of the protein and the complexes are reduced (Fig. 1B and D). While KREPA3 with the much larger TAP-tag also gets incorporated into editosomes without a reduction in overall abundance, a significant amount of editosome subcomplexes are generated [49]. Mutation of the KREPA3 ZFs also reduces the abundance of KREPA3 protein as well as entire editosomes. Indeed, exclusive expression of KREPA3 with a myc tag and mutated ZF1 or ZF1&2 results in essentially complete loss of KREPA3 (Fig. 1B) and editosomes (Data not shown). Exclusive expression of KREPA3myc with mutation of ZF2 also changes the relative abundance of KREPA1:KREPA2:KREL1 compared to the cells expressing KREPA3 WT (Fig. 1D). Hence, the ZFs of KREPA3 are important for the structural integrity of editosomes.

### Editosome Stability and Structural Integrity

KREPA3 is essential to editosome stability and structural integrity [49] but the precise nature of this role is only partially understood. The absence of the other editosome proteins is probably due to their degradation as a consequence of not being integrated into editosomes during assembly, or not retained in unstable or disrupted editosomes. How editosome proteins are targeted for degradation in the absence of stable complex formation is not yet understood. The OB fold and ZFs domains of KREPA3 are involved in editosome structural integrity and stability. Exclusive expression of TAP tagged KREPA3 that is devoid of an OB fold results in the virtual absence of 20S complexes [49]. Editosomes are not lost but are reduced in abundance upon exclusive expression of myc tagged KREPA3 with mutated ZF2 (Fig. 1B and D) or of TAP-tagged KREPA3 with mutated ZF1 and/or ZF2 [49]. In addition, mutated ZF2 alters the protein stoichiometries of the ∼20S editosome. The relative abundance of KREPA1 was reduced compared to KREPA2 and KREL1 in ∼20S editosomes (Fig. 1D). As described in the introduction, the KREPA1 binds to KRET2 and KREL2 to form the insertion subcomplex, while the KREPA2 similarly binds to KREX2 and KREL1 to form the deletion subcomplex [18]. The relative loss of KREPA1 from ∼20S editosomes after exclusive expression of KREPA3 with mutated ZF implies an involvement of the ZF motifs in the association of the insertion subcomplex in the ∼20S editosome. An alternative hypothesis is that the components of the deletion subcomplex are less susceptible to degradation than the components of the insertion subcomplex.

The different effects attributed to the use of TAP or myc tags complicate analysis of KREPA3 mutations. WT myc-tagged KREPA3 was incorporated into ∼20S editosomes and had no effect on cell growth or RNA editing (Fig. 1A to D), but simultaneous expression of untagged KREPA3 resulted in the relatively poor accumulation of myc-tagged KREPA3, presumably because myc-tagged KREPA3 is less efficiently incorporated into or retained by editosomes than the untagged protein. Not surprisingly, the larger TAP tag was even more disruptive and resulted in subcomplexes and inhibited growth and RNA editing to some extent [49]. By contrast, almost all of the myc tagged KREPA3 is in ∼20S editosomes under these conditions (Fig. 1D). In addition, the greater abundance of the TAP tagged protein suggests it is more stable in comparison to the myc tagged protein under conditions where the wild type protein is also expressed; indeed the myc tagged protein is essentially absent (Fig. 1B versus [49] Fig. 5C). The inability of myc-tagged KREPA3 with mutated ZF2 to rescue cells upon inactivation of expression of WT KREPA3 Reg allele (Fig. 1A) shows that the ZFs have essential functions in vivo. However, the ZF2 mutation in both cases results in a proportional reduction of insertion subcomplexes, for example seen as a reduction of KREPA1 (Fig. 1D versus [49] Fig. 7A). Overall, these results are consistent with TAP tagged KREPA3 being more disruptive and more stable while myc tagged KREPA3 being less disruptive and less stable and the ZFs having a role in association with the insertion subcomplex. These results could imply that KREPA3 is involved in the association of subcomplexes within ∼20S editosomes, the aggregation of subcomplexes, or some dynamic process of editing, such as protein exchange that has been affected by the ZF2 mutation.

Limited knowledge exists about the internal organization of ∼20S editosoems and the interactions among its proteins, and even less known about specific interactions between particular proteins. In general, ∼20S editosomes can be divided into the two heterotrimeric insertion and deletion subcomplexes, along with several other proteins including the endonucleases, plus several related KREPA proteins, including KREPA3 [18]. The KREPA family of proteins includes KREPA1 and KREPA2, which are integral components of the insertion and deletion subcomplexes, respectively [46], and KREPA3-6. The KREPA family of proteins provides a network of interactions among each other and with other editosome proteins that positions the various functional domains of the editosome proteins [61]. The KREPA3, 4, 5, and 6 proteins appear particularly essential for editosome integrity since knockdown of KREPA3, 4, or 6 expression results in total loss of editosomes [49]–[53]. KREPA5 has not yet been tested, but its sequence similarity to A4 and A6 suggest a similar role in editosome function. KREPA3 has two ZF and an OB fold motif, as do KREPA1 and KREPA2, and such motifs have been variously reported in many systems to function in protein/protein or protein/nucleic acid interactions depending on the protein [62–65]. The results herein imply that these motifs function in protein/protein interaction since mutation of the ZFs or elimination of the OB fold affect editosome structural integrity. Although RNA is not essential for editosome integrity [66], these results do not exclude the possibility that KREPA3 also interacts with RNA.

### Specific Functional Roles of KREPA3

The ZFs of KREPA3 may function in a way that affects editing either directly or indirectly. If the ZFs function in binding to other proteins, as suggested above, their role may be to structurally organize editosomes so that functional domains of proteins are properly positioned relative to the RNA substrates. Interactions of KREPA3 with other editosome proteins might affect their activity much as the interaction of KREPA1 and KREPA2 with their binding partners affected their catalytic activities [46]. Hence, the ZF mutations may indirectly affect editing by altering the positions of editosome protein functional domains and perhaps their activities. Alternatively, since we cannot exclude the possibility that the ZFs or other domains of KREPA3 bind RNA, it may function by positioning the substrate RNAs relative to the functional domains of the other editosome proteins. Hence, the ZF mutations might directly affect editing by mis-positioning the RNA relative to the functional domains of the editosome proteins. The differential consequences of mutations of ZF1 vs ZF2 (Fig. 1B, and 2 to [Fig pone-0008913-g003] [Fig pone-0008913-g004] 5) might reflect differential affinity for editosome proteins or the RNA substrates. The two C2H2 ZF motifs in KREPA3 have different amino acid compositions and spacing [47]. The differential effects of the mutation of these two ZFs on the extent of A6 mRNA editing (Fig. 3 and 5) may be due to different specific binding characteristics of the ZFs. These different ZF mutations could result in differential interaction with other editosome proteins which might affect their conformation, catalytic activities, and/or interaction with RNA substrates; they could also impair direct interactions between KREPA3 and RNA substrates in distinct ways. The C-terminal OB-fold of KREPA3 may also have RNA binding activity similar to other proteins with OB folds [65]. Because this region functions in interaction with other editosome proteins (A. Schnaufer, unpublished data), the absence of ∼20S editosomes in cells solely expressing KREPA3 from which the C-terminal OB fold was deleted is not surprising [49]. However, editing is a dynamic process that involves coordinated catalytic steps, and typically the use of multiple gRNAs [67]. Hence it is likely that there is significant movement of the substrate RNAs relative to the catalytic centers as well as realignment of mRNA and gRNA interactions during the process. Thus, the ZF and OB fold domains of KREPA3 may undergo a series of coordinated transient interactions with the other editosome proteins and the RNA substrates during the steps of editing. The ZFs may enhance editing efficiency by optimizing editosome organization, RNA interaction, and dynamic movements.

### Accuracy of Editing

The partially edited A6 mRNAs that accumulate in cells that exclusively express KREPA3 with mutated ZF2 (Fig. 3) resemble those that are present at much lower frequency in wild type cells. They may represent intermediates in the process of RNA editing or non-functional erroneous end-products although some might be alternative-edited mRNAs that generate diverse proteins with novel functions [68]–[70]. At present, it is difficult to discern the role these partially edited mRNAs have in vivo. Such partially edited mRNAs might be a consequence of T. brucei encoding thousands of different gRNAs, many more than minimally needed for the editing, many of which specify substantially overlapping regions. However, L. tarentolae which has little gRNA diversity also contains such partially edited mRNAs [58], so the partially edited mRNA can not be only due to gRNA diversity. Precise uridine insertion and deletion is necessary to generate the conventional fully edited mRNA and hence functional protein. Currently, little is known whether the partially edited mRNAs which contain open reading frames can be translated into proteins. The occurrence of the diverse partially edited mRNAs could result from a combination of factors including gRNA diversity and redundancy, compositionally distinct editosomes with different endonuclease specificities, and the diversity of editing accessory proteins and complexes. The precise coordination of these factors is the prerequisite for “accurate” editing to create the fully edited mRNAs. Hence, the accumulation of partially edited RNAs due to mutation of ZF2 or the virtual loss of editing due to the mutation of ZF1 may reflect the role of these ZFs in ensuring accurate and efficient editing and perhaps the transition from one gRNA to the next. Nevertheless, both insertion and deletion editing occurs despite the ZF mutations. Hence, the KREPA3 ZF does not appear to be essential for discrimination between such sites. After demonstrating that editing requires three distinct editosomes, Carnes et al [20] proposed alternative models for editing at different sites: site specific use of compositionally stable editosomes or physical exchange of the different endonucleases within a stable “core” complex. These models have different implications for how the number and type of editosomes must associate with the mRNA-gRNA substrates to accomplish editing of insertion and deletion sites within the same substrate, e.g. one editosome per substrate vs multiple different editosomes. For both models, the ZF and OB fold domains of KREPA3 may have critical roles in coordinating the catalytic and RNA translocation steps of editing.

The results presented here also indicate that ∼20S editosomes are not required for the processing and maturation of gRNAs since mature gRNAs are generated in normal amounts in the absence of editosomes (Fig. 6). Hence, the complexes that are associated with the processing of gRNAs and sediment at ∼20S as do editosomes [71] must be different complexes.

Compared to our previous data, which showed that KREPA3 ZF mutation had no effect on in vitro editing activities but dramatically reduced the fully edited mRNAs level in vivo [49], we showed here that the defect in editing in vivo caused by KREPA3 ZF mutation results from an inhibition of editing progression. This study also shows that the two ZFs of KREPA3 likely play different roles in editing process. Because the myc-tag, unlike the TAP-tag, does not alter KREPA3 integration into the editosome, we could demonstrate that ZF mutations themselves do not completely disrupt editosome integrity, although mutation to ZF2 does alter editosome stoichiometry. In addition, we give the first demonstration that the editosome is not involved in the processing and maturation of gRNAs, because loss of editosomes did not affect total gRNA level. These new insights will be helpful in understanding the editing process in vivo.

## Materials and Methods

### Plasmid Construction and Transfection

The previously generated pLEW79-TAP plasmids with TAP-tagged KREPA3 WT and ZF mutant genes [49] contain a C-myc tag gene located between KREPA3 and TAP and adjacent to the downstream of KREPA3 gene. Myc-tagged full length KREPA3 WT and mutant genes with one or both zinc fingers mutation were amplified from the plasmids pLEW79-A3-TAP, pLEW79-A3ZFm1-TAP, pLEW-A3ZFm2-TAP and pLEW79-A3ZFm1&2-TAP [49], respectively, by using the primers 5′-CCTCGAGCCACCATGAAGCGTGTTACTTCAC-3′ and 5′-ATTCATGATCACAGGTCTTCTTCAGAGATCAG-3′. After digestion with *Xho*I and *Bcl*I (restriction sites are underlined), the PCR products were inserted into pHD1344tub to create pHD1344-A3-myc, pHD1344-A3ZFm1-myc, pHD-1344-A3ZFm2-myc and pHD1344-Z3ZFm1&2-myc plasmids. These plasmids were linearized with *Not*I and transfected as described previously [27] into *T. brucei* BF KREPA3-RKO cells [49] independently, and the cells were grown in HMI-9 medium with 10% FBS containing 2.5 µg/ml G418, 5 µg/ml hygromycin B, 1 µg/ml tetracycline and 2.5 µg/ml phleomycin at 37°C. Integration of pHD1344tub is targeted to the β-tubulin locus, where constitutive expression of the introduced alleles is driven by readthrough transcription. After selecting with 0.1 µg/ml of puromycin/ml, the resulting clones were designated RKO-A3 WT-myc, ZFm1-myc, ZFm2-myc, and ZFm1&2-myc, respectively. Expression of the tagged genes was determined by Western analysis. The expression of the KREPA3 Reg allele was repressed by culturing the cells in medium minus tet. Growth of the cells was monitored in the presence or absence of tet and diluted to 1.0×10^5^ to 2.0×10^5^ cells/ml.

### RNA Isolation and RT-PCR Analysis

Total RNA was harvested from the cell lines using the TRIzol reagent (Gibco-BRL) according to manufacturer's instructions. 10 µg of RNA was treated with DNase I by using a DNA-free kit (Ambion) and used as the templates for RT-PCR analysis. Real-time RT-PCR to measure the relative abundance of mitochondrial mRNAs was performed as previously described [19]. RT-PCR analysis of RNA editing, which is used to amplify the pre-edited and all the edited mRNAs simultaneously, was performed as previously described [27]. Briefly, 1 µg of DNase I treated RNA was annealed to 75 pmol of downstream primer by incubation at 70°C for 5 min and cooling slowly. The annealed primer was extended with Superscript III Reverse transcriptase (Invitrogen) for 1 hr at 42°C. 75 pmol of upstream primer was added and PCR was performed. PCR products of A6 mRNA and MURF2 and ND4 mRNAs were analyzed on 1.2% and 2% agarose gel, respectively. The band was cut and cloned into pGEM-T easy vector (Promega), and the inserts were sequenced by standard procedures using SP6 or T7 promoter primer. The upstream and downstream primers used for RT-PCR analysis were: 5′-AAAAATAAGTATTTTGATATTATTAAAG-3′ and 5′-TATTATTAACTTATTTGATC-3′ for A6 mRNA, 5′-ATAGAAAGGTATATAATCTATAATG-3′ and 5′-AATATAAAATCTAGATCAAACCATCACA-3′ for MURF2, 5′-TGTGTGACTACCAGAGAT-3′ and 5′-ATCCTATACCCGTGTGTA-3′ for ND4 mRNA.

### Glycerol Gradient Sedimentation and Western Analysis

Crude mitochondria were prepared from 3×10^9^ BF KREPA3-RKO in the presence or absence of 1 µg/ml tet or from RKO-A3 WT-myc or ZFm2-myc cells witout tet induction as previously described [49]. After lysis in 600 µl mt lysis buffer (20 mM HEPES [pH 7.9], 10 mM magnesium acetate, 100 mM KCl, 1 mM EDTA) and centrifugation at 13,000 rpm for 10 min at 4°C, the cleared lysates were loaded onto 4.5-ml 10–30% glycerol gradients and centrifuged at 44,000 rpm for 5 h at 4°C in a SW55 rotor (Beckman). 12 fractions of 460 µl were collected from top to bottom and flash frozen in liquid nitrogen, and then stored at −80°C. For each fraction, 30 µl was resolved on 12% SDS-PAGE gel and transferred to PVDF membrane (Immobilon-P, Millipore) for western analysis. The membrane was first blocked by incubating in 5% non-fat milk for 1 hour, and then probed with a cocktail of MAbs against KREPA1, KREPA2, KREL1, and KREPA3, followed by HRP-conjugated goat anti-mouse IgG secondary (Bio-Rad), and finally visualized by chemiluminescence (ECL Pierce) and exposure to X-ray film.

### gRNA Capping Assay

Total RNA was extracted from KREPA3-RKO, RKO-A3 WT-myc and ZFm2-myc cells with KREPA3 Reg allele expressed (E) or repressed (R), and then treated with DNase I as described above. 1 µg of treated RNA was incubated at 37°C for 1 h in a 15 µl reaction containing 40 µCi [α-^32^P]GTP (3000 Ci/mmol) and 10U of guanylyltransferase (Epicentre Biotechnologies) according to the manufacturer's instructions. The reaction products were separated by electrophoresis on 10% polyacrylamide gel containing 7M urea and 1X TBE, transferred to Whatman paper, dried, and then visualized after exposure to PhosphorImager screen (GE Healthcare). Labeled gRNAs were identified by size in comparison to the labeled low range ssRNA ladder (NEB).

## Supporting Information

Figure S1 Lower level of KREPA3 is sufficient for cell growth and RNA editing. The expression level of KREPA3 Reg protein was regulated by adjusting tet concentration to 1000, 5, 4, 3, 2, 1, and 0 ng/ml, respectively, in KREPA3-RKO cells. (A) Growth of KREPA3-RKO cells at different tet concentrations. The cells grew normally when tet concentration was 3 ng/ml or more, but was inhibited obviously at 2 ng/ml and dramatically at 1 ng/ml. (B) Western analysis of KREPA3 protein level at different tet concentrations or non-induced at day 3 by using MAb against KREPA3. The expression from KREPA3 Reg allele was reduced dramatically when tet was adjusted from 1000 ng/ml to 5 ng/ml and was undetectable when tet was 2 ng/ml or lower. (C) RT-PCR products of A6 and MURF2 mRNAs from KREPA3-RKO cells induced with different tet concentrations or non-induced at day 3. The pre-edited and edited products were indicated. Progressive decreases in KREPA3 Reg expression resulted in concomitant decreases in the levels of partially and fully edited A6 mRNAs and fully edited MURF2 mRNAs, while the pre-edited mRNAs of both A6 and MURF2 accumulated dramatically. When KREPA3 protein is undetectable, the edited mRNAs were subsequently eliminated.
(0.66 MB TIF)

Figure S2 Sequence and location of gMURF2-I and gMURF2-II genes in *T. brucei*. (A) Editing of MURF2 mRNA is mediated by two gRNAs. (B) Both gMURF2-I and gMURF2-II are transcribed from maxicircle (18, 62).
(0.32 MB TIF)

Figure S3 TAP-tagged KREPA3 ZF mutants showed the same effect on RNA editing as the myc-tagged ones. RT-PCR products of A6 mRNAs from KREPA3-RKO cells exclusively expressing either TAP-tagged or myc-tagged KREPA3 WT and ZFm2 (R) were analyzed by agarose gel electrophoresis. The products from KREPA3-RKO cells with KREPA3 Reg expressed (E) and repressed (R) were run as control. The pre-edited, partial-edited and edited products of A6 are indicated. Exclusive expression of TAP-tagged KREPA3ZFm2 showed the same effect on the editing of A6 as myc-tagged KREPA3ZFm2: the disappearance of the fully edited A6 and accumulation of some partially edited species, especially the one close to the pre-edited band.
(0.25 MB TIF)
