# Supplementary figures and images for: The Zinc-Fingers of KREPA3 Are Essential for the Complete Editing of Mitochondrial mRNAs in Trypanosoma brucei
Source: PLoS One. 2010 Jan 27;5(1):e8913. doi: 10.1371/journal.pone.0008913 (PMC2811742; doi:10.1371/journal.pone.0008913)

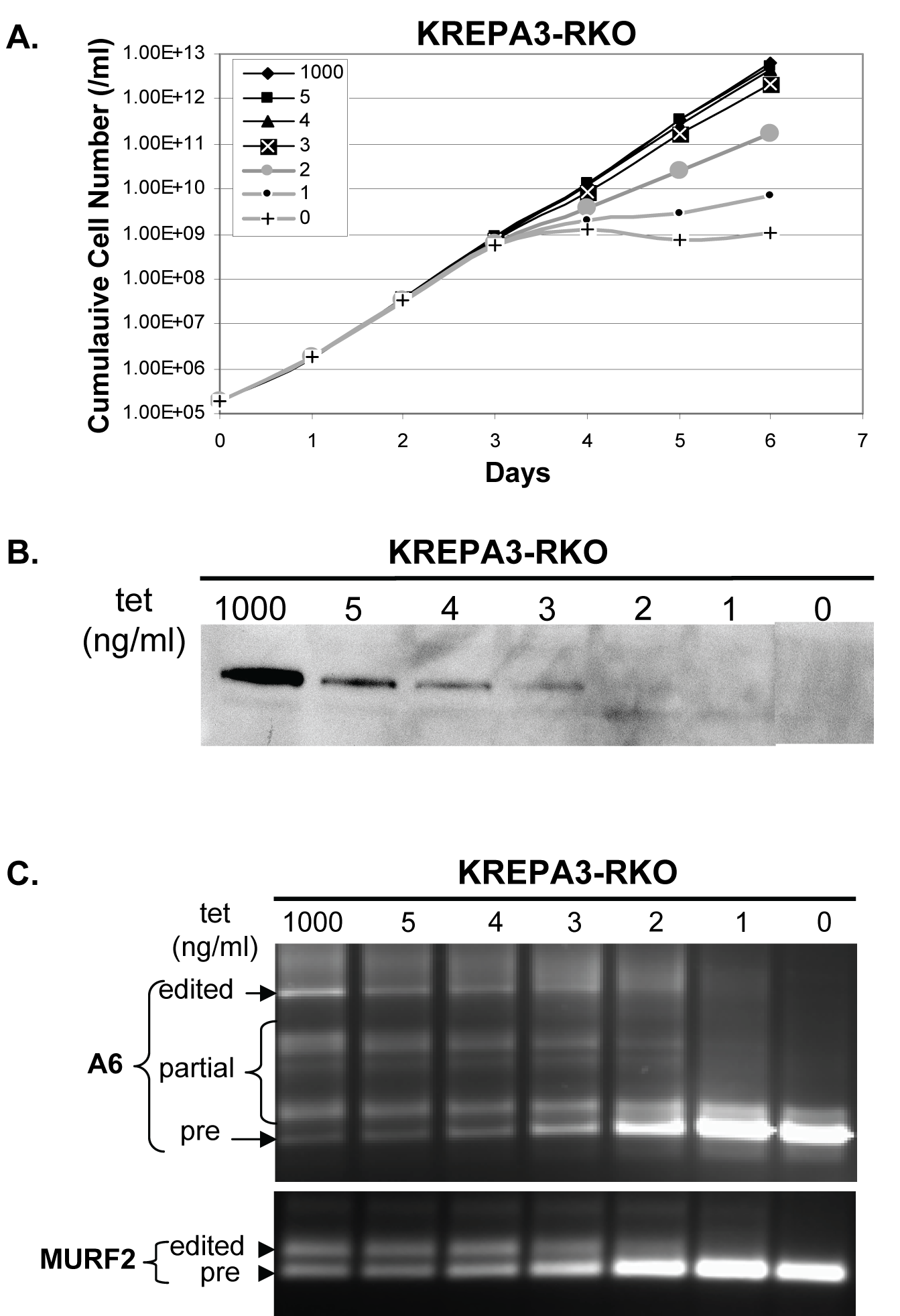

Supplement: Figure S1 — Lower level of KREPA3 is sufficient for cell growth and RNA editing. The expression level of KREPA3 Reg protein was regulated by adjusting tet concentration to 1000, 5, 4, 3, 2, 1, and 0 ng/ml, respectively, in KREPA3-RKO cells. (A) Growth of KREPA3-RKO cells at different tet concentrations. The cells grew normally when tet concentration was 3 ng/ml or more, but was inhibited obviously at 2 ng/ml and dramatically at 1 ng/ml. (B) Western analysis of KREPA3 protein level at different tet concentrations or non-induced at day 3 by using MAb against KREPA3. The expression from KREPA3 Reg allele was reduced dramatically when tet was adjusted from 1000 ng/ml to 5 ng/ml and was undetectable when tet was 2 ng/ml or lower. (C) RT-PCR products of A6 and MURF2 mRNAs from KREPA3-RKO cells induced with different tet concentrations or non-induced at day 3. The pre-edited and edited products were indicated. Progressive decreases in KREPA3 Reg expression resulted in concomitant decreases in the levels of partially and fully edited A6 mRNAs and fully edited MURF2 mRNAs, while the pre-edited mRNAs of both A6 and MURF2 accumulated dramatically. When KREPA3 protein is undetectable, the edited mRNAs were subsequently eliminated. (0.66 MB TIF) [file pone.0008913.s001.tif]

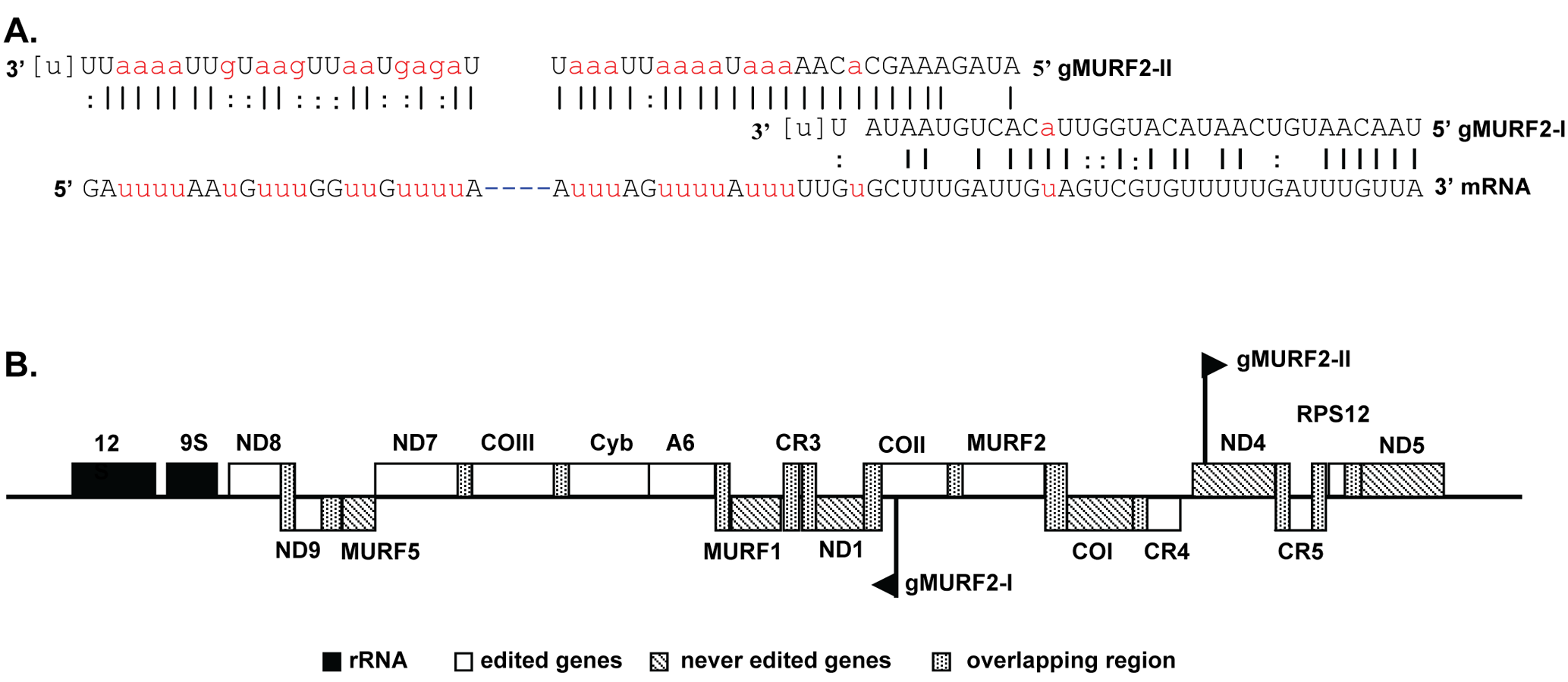

Supplement: Figure S2 — Sequence and location of gMURF2-I and gMURF2-II genes in T. brucei. (A) Editing of MURF2 mRNA is mediated by two gRNAs. (B) Both gMURF2-I and gMURF2-II are transcribed from maxicircle (18, 62). (0.32 MB TIF) [file pone.0008913.s002.tif]

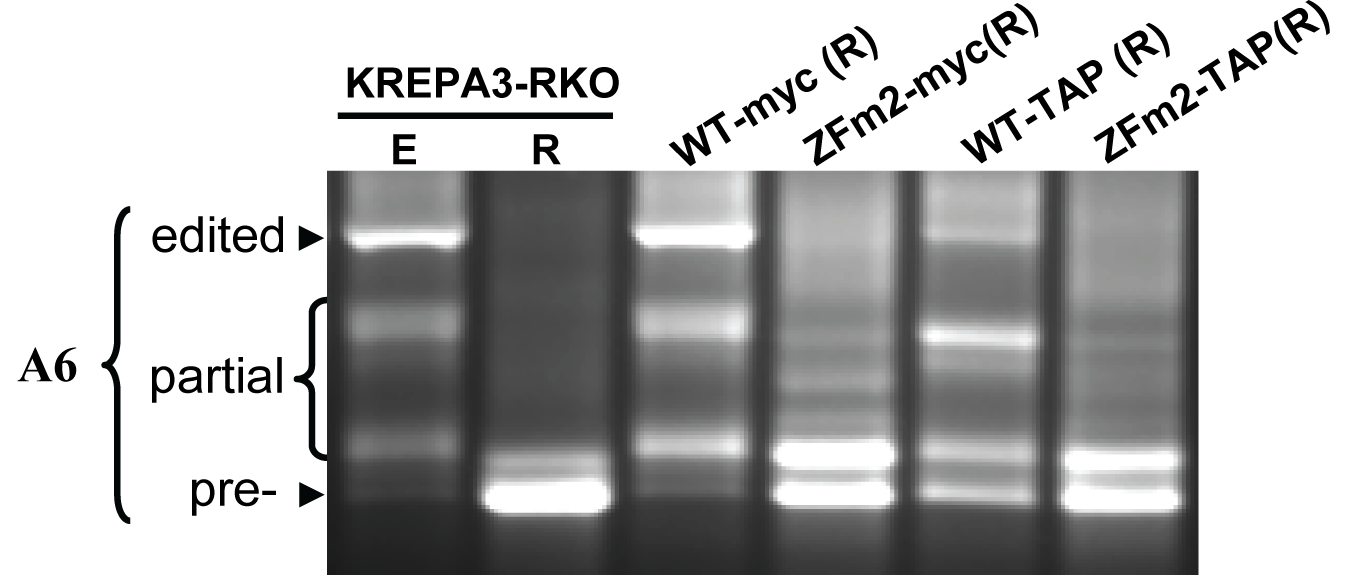

Supplement: Figure S3 — TAP-tagged KREPA3 ZF mutants showed the same effect on RNA editing as the myc-tagged ones. RT-PCR products of A6 mRNAs from KREPA3-RKO cells exclusively expressing either TAP-tagged or myc-tagged KREPA3 WT and ZFm2 (R) were analyzed by agarose gel electrophoresis. The products from KREPA3-RKO cells with KREPA3 Reg expressed (E) and repressed (R) were run as control. The pre-edited, partial-edited and edited products of A6 are indicated. Exclusive expression of TAP-tagged KREPA3ZFm2 showed the same effect on the editing of A6 as myc-tagged KREPA3ZFm2: the disappearance of the fully edited A6 and accumulation of some partially edited species, especially the one close to the pre-edited band. (0.25 MB TIF) [file pone.0008913.s003.tif]
